# Supplementary material for: Machine learning based prediction of lattice thermal conductivity for half-Heusler compounds using atomic information
Source: Sci Rep. 2021 Jun 28;11:13410. doi: 10.1038/s41598-021-92030-4 (PMC8239013; doi:10.1038/s41598-021-92030-4)
Supplement: Supplementary file 1 — Supplementary Informations. [file 41598_2021_92030_MOESM1_ESM.docx]

Supplemental Material

**Machine Learning based Prediction of Lattice Thermal Conductivity for Half-Heusler Compounds using Atomic Information**

Hidetoshi Miyazaki^1, 2*^, Tomoyuki Tamura^1, 3^, Masashi Mikami^4^, Kosuke Watanabe^1#^, Naoki Ide^1^, Osman Murat Ozkendir^5^, and Yoichi Nishino^1^

^1^ *Nagoya Institute of Technology, Dept. of Physical Science and Engineering, 466-8555, Nagoya, Japan*

^2^*Frontier Research Institute for Materials Science, Nagoya Institute of Technology, Gokiso-cho, Showa-ku, Nagoya 466-8555, Japan*

^3^ *Center for Materials research by Information Integration, National Institute for Materials Science (NIMS), Tsukuba 305-0047, Japan*

^4^ *National Institute of Advanced Industrial Science and Technology, 2266-98 Anagahora, Shimoshidami, Moriyama, Nagoya 463-8560*

^5^ *Department of Natural and Mathematical Sciences, Faculty of Engineering, Tarsus University, 33400, Tarsus, Turkey*

*miyazaki@nitech.ac.jp

^#^Present address : *Department of Mechanical and Control Engineering, Kyushu Institute of Technology, 1-1 Sensui, Tobata-ku, Kitakyushu, Fukuoka 804-8550, Japan*

Figure S1 shows the periodic table of the site selection system of the constituent elements for the 143 half-Heusler compounds. The elements from groups 8 to 11 occupy almost 4*c* sites; groups 1 to 6 and the lanthanide and actinide series occupy 4*a* sites and some elements also occupy 4*b* sites. The elements of groups 12–16 occupy 4*b* sites as well as 4*c* sites for most of the elements. This suggests that the site selection in half-Heusler compounds is not determined solely by the relationship between the atomic radii of the elements at each site but also by the atomic masses at each site, which is a complex balance of the two elemental information.


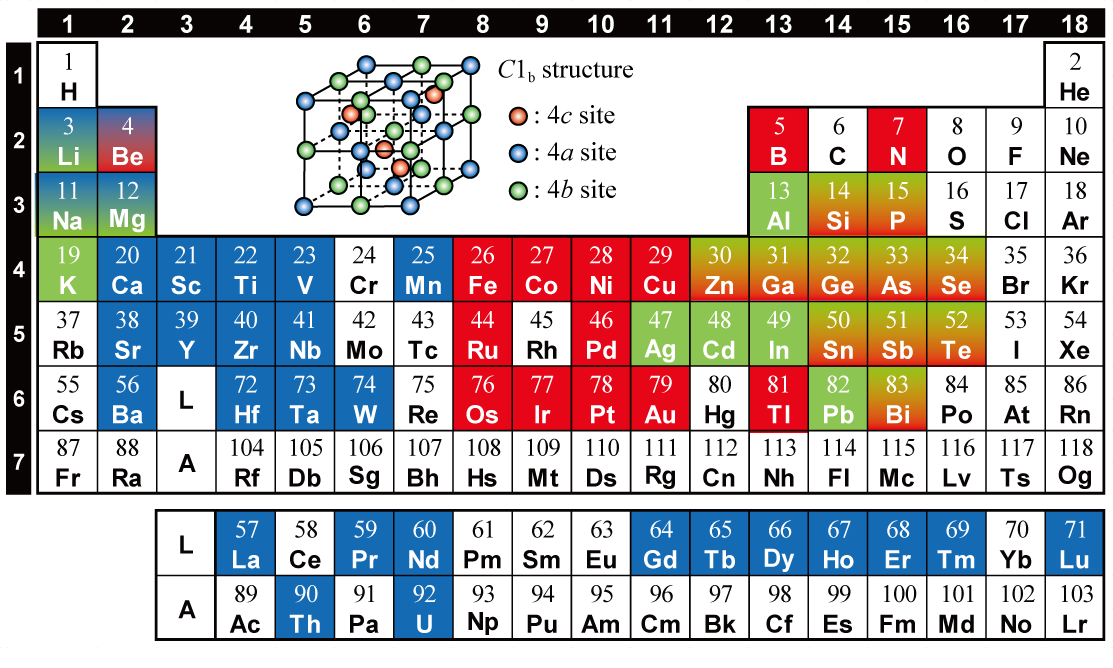


Figure S1

Periodic table summarizing the site-selective system for each element in the half-Heusler compound determined by DFT calculations.

Table S1

Table of the Materials ID, Chemical formula, optimal site locations, lattice parameters, and thermal conductivities for 143 half-Heusler compounds. The atoms occupying each site and the corresponding lattice parameter are shown for the optimal site selection with the lowest total energy. Thermal conductivities are calculated using the lattice thermal conductivity calculation code Phono3py [1] for the best site combination.

| Materials ID | Chemical Fomula | 4*c* site | 4*a* site | 4*b* site | L. P. | T. C. |  | Materials ID | Chemical Fomula | 4*c* site | 4*a* site | 4*b* site | L. P. | T. C. |
| --- | --- | --- | --- | --- | --- | --- | --- | --- | --- | --- | --- | --- | --- | --- |
| 2894 | ScSnAu | Au | Sc | Sn | 0.653 | 7.09 |  | 20269 | MnGaPt | Pt | Mn | Ga | 0.600 | 2.33 |
| 3161 | LiAlSi | Si | Li | Al | 0.594 | 5.90 |  | 20415 | GdPbAu | Au | Gd | Pb | 0.689 | 3.18 |
| 3432 | ScNiSb | Ni | Sc | Sb | 0.612 | 13.91 |  | 20514 | GdSnAu | Au | Gd | Sn | 0.679 | 3.73 |
| 3462 | TmSnAu | Au | Tm | Sn | 0.669 | 4.61 |  | 20952 | TiNiSb | Ni | Ti | Sb | 0.596 | 3.64 |
| 3522 | MgCuSb | Cu | Mg | Sb | 0.625 | 3.68 |  | 21272 | ErNiSb | Ni | Er | Sb | 0.633 | 9.68 |
| 3716 | TbNiSb | Ni | Tb | Sb | 0.639 | 9.06 |  | 21425 | UNiSn | Ni | U | Sn | 0.636 | 5.36 |
| 4025 | TmNiSb | Ni | Tm | Sb | 0.631 | 9.85 |  | 22786 | ThNiSn | Ni | Th | Sn | 0.660 | 7.15 |
| 4174 | HoNiSb | Ni | Ho | Sb | 0.635 | 9.69 |  | 30377 | ErPbAu | Au | Er | Pb | 0.682 | 3.51 |
| 4262 | BeAlB | B | Be | Al | 0.496 | 18.71 |  | 30389 | HoPbAu | Au | Ho | Pb | 0.684 | 3.43 |
| 4510 | DyNiSb | Ni | Dy | Sb | 0.636 | 9.46 |  | 30390 | HoSnAu | Au | Ho | Sn | 0.673 | 4.46 |
| 4964 | YSbPt | Pt | Y | Sb | 0.663 | 3.23 |  | 30413 | TbPbAu | Au | Tb | Pb | 0.687 | 3.26 |
| 5177 | LuSnAu | Au | Lu | Sn | 0.666 | 4.72 |  | 30453 | DyBiPt | Pt | Dy | Bi | 0.675 | 6.23 |
| 5640 | ErSnAu | Au | Er | Sn | 0.671 | 4.53 |  | 30454 | ErBiPt | Pt | Er | Bi | 0.672 | 6.35 |
| 5920 | LiAlGe | Ge | Li | Al | 0.601 | 5.08 |  | 30455 | HoBiPt | Pt | Ho | Bi | 0.674 | 6.46 |
| 5967 | TiCoSb | Co | Ti | Sb | 0.590 | 22.13 |  | 30457 | LuNiBi | Ni | Lu | Bi | 0.641 | 9.83 |
| 7173 | ScSbPt | Pt | Sc | Sb | 0.640 | 7.67 |  | 30459 | ScNiBi | Ni | Sc | Bi | 0.627 | 9.06 |
| 7575 | LiZnN | N | Li | Zn | 0.493 | 17.39 |  | 30460 | YNiBi | Ni | Y | Bi | 0.651 | 5.91 |
| 9124 | LiZnAs | As | Li | Zn | 0.598 | 13.89 |  | 30847 | TiSnPt | Pt | Ti | Sn | 0.624 | 11.18 |
| 9437 | NbFeSb | Fe | Nb | Sb | 0.596 | 23.43 |  | 30848 | USnPt | Pt | U | Sn | 0.667 | 5.41 |
| 10194 | LuSbPt | Pt | Lu | Sb | 0.655 | 6.42 |  | 31451 | ZrCoBi | Co | Zr | Bi | 0.624 | 17.68 |
| 10687 | LiCdP | P | Li | Cd | 0.614 | 20.07 |  | 31454 | TaSbRu | Ru | Ta | Sb | 0.620 | 16.23 |
| 11242 | DyPbAu | Au | Dy | Pb | 0.685 | 3.35 |  | 31455 | VSbRu | Ru | V | Sb | 0.605 | 6.90 |
| 11520 | YNiSb | Ni | Y | Sb | 0.638 | 9.03 |  | 31457 | ZrSbRu | Ru | Zr | Sb | 0.636 | 3.36 |
| 11836 | ErSbPd | Pd | Er | Sb | 0.657 | 7.65 |  | 36111 | LiMgP | P | Li | Mg | 0.600 | 19.94 |
| 11839 | GdSbPt | Pt | Gd | Sb | 0.666 | 3.34 |  | 505297 | NbSbRu | Ru | Nb | Sb | 0.620 | 15.65 |
| 11869 | HfSnPd | Pd | Hf | Sn | 0.637 | 13.99 |  | 567418 | HoSbPd | Pd | Ho | Sb | 0.659 | 7.72 |
| 12558 | LiMgAs | As | Li | Mg | 0.620 | 20.18 |  | 567422 | GdNiBi | Ni | Gd | Bi | 0.653 | 8.12 |
| 13308 | HoGeAu | Au | Ho | Ge | 0.653 | 4.34 |  | 567636 | VFeSb | Fe | V | Sb | 0.580 | 14.20 |
| 16313 | TbSbPt | Pt | Tb | Sb | 0.664 | 3.82 |  | 568269 | TmNiBi | Ni | Tm | Bi | 0.644 | 9.36 |
| 16314 | TmSbPt | Pt | Tm | Sb | 0.657 | 5.86 |  | 569197 | GdNiSb | Ni | Gd | Sb | 0.641 | 8.38 |
| 16327 | DySbPt | Pt | Dy | Sb | 0.662 | 4.43 |  | 569779 | ScSbPd | Pd | Sc | Sb | 0.639 | 11.07 |
| 16329 | ErSbPt | Pt | Er | Sb | 0.659 | 5.41 |  | 570213 | LiMgBi | Bi | Li | Mg | 0.682 | 15.77 |
| 16376 | HoSbPt | Pt | Ho | Sb | 0.661 | 4.95 |  | 620271 | GdBiPt | Pt | Gd | Bi | 0.679 | 5.54 |
| 19886 | ThSnPt | Pt | Th | Sn | 0.683 | 4.15 |  | 621592 | YPbAu | Au | Y | Pb | 0.687 | 3.28 |
| 20185 | LuNiSb | Ni | Lu | Sb | 0.628 | 9.97 |  | 924128 | HfNiSn | Ni | Hf | Sn | 0.612 | 16.44 |
| 924129 | ZrNiSn | Ni | Zr | Sn | 0.616 | 19.05 |  | 1076916 | GdBiPd | Pd | Gd | Bi | 0.677 | 6.80 |
| 924130 | TiNiSn | Ni | Ti | Sn | 0.596 | 17.54 |  | 1093991 | ZrAsIr | Ir | Zr | As | 0.618 | 10.43 |
| 961646 | TiTeOs | Os | Ti | Te | 0.618 | 12.96 |  | 1094088 | NbCoSn | Co | Nb | Sn | 0.597 | 18.62 |
| 961649 | ZrFeTe | Fe | Zr | Te | 0.610 | 20.00 |  | 1100391 | NaBeSb | Be | Na | Sb | 0.625 | 3.62 |
| 961653 | FeSiW | Fe | Si | W | 0.556 | 6.21 |  | 1100392 | LiSiB | B | Li | Si | 0.501 | 11.09 |
| 961657 | YNiP | Ni | Y | P | 0.598 | 2.37 |  | 1100393 | LiAgSe | Se | Li | Ag | 0.628 | 2.72 |
| 961659 | TiSiPd | Pd | Ti | Si | 0.589 | 25.31 |  | 1100403 | KBaSb | Sb | K | Ba | 0.828 | 2.88 |
| 961660 | TiFeSe | Fe | Ti | Se | 0.562 | 27.60 |  | 1100404 | VAsRu | Ru | V | As | 0.580 | 5.04 |
| 961661 | ZrSiPd | Pd | Zr | Si | 0.612 | 18.35 |  | 1100409 | LiCaSb | Sb | Li | Ca | 0.712 | 26.07 |
| 961665 | MgScGa | Ga | Mg | Sc | 0.647 | 3.88 |  | 1100411 | CaCdSn | Sn | Ca | Cd | 0.712 | 3.02 |
| 961673 | TiFeTe | Fe | Ti | Te | 0.589 | 20.30 |  | 1100412 | TiTeRu | Ru | Ti | Te | 0.615 | 14.54 |
| 961675 | ScNiP | Ni | Sc | P | 0.569 | 8.87 |  | 1100420 | NaCaSb | Sb | Na | Ca | 0.740 | 5.45 |
| 961678 | ScCoTe | Co | Sc | Te | 0.607 | 12.70 |  | 1100424 | LiMgSb | Sb | Li | Mg | 0.667 | 14.63 |
| 961682 | TiSnPd | Pd | Ti | Sn | 0.622 | 11.03 |  | 1100425 | LiCdSb | Sb | Li | Cd | 0.674 | 3.51 |
| 961684 | LiCaAs | As | Li | Ca | 0.667 | 20.16 |  | 1100429 | KSrSb | Sb | K | Sr | 0.804 | 3.66 |
| 961685 | NaCaAs | As | Na | Ca | 0.696 | 7.57 |  | 1100430 | LiZnSb | Zn | Li | Sb | 0.632 | 4.81 |
| 961687 | ZrSnPd | Pd | Zr | Sn | 0.641 | 14.04 |  | 1100433 | CaZnSi | Si | Ca | Zn | 0.648 | 2.46 |
| 961693 | ZrInAu | Au | Zr | In | 0.653 | 9.42 |  | 1100436 | LiInSi | Si | Li | In | 0.628 | 3.61 |
| 961697 | ScGeAu | Au | Sc | Ge | 0.630 | 12.52 |  | 1206667 | PrNiBi | Ni | Pr | Bi | 0.668 | 4.47 |
| 961698 | LiZnP | P | Li | Zn | 0.576 | 9.72 |  | 1206679 | YBiPt | Pt | Y | Bi | 0.676 | 4.61 |
| 961706 | TiSiPt | Pt | Ti | Si | 0.591 | 34.51 |  | 1206681 | ScBiPd | Pd | Sc | Bi | 0.653 | 5.85 |
| 961711 | ZrSiPt | Pt | Zr | Si | 0.615 | 14.11 |  | 1206686 | TbNiBi | Ni | Tb | Bi | 0.651 | 8.57 |
| 961713 | ZrSnPt | Pt | Zr | Sn | 0.643 | 13.23 |  | 1206712 | ErNiBi | Ni | Er | Bi | 0.646 | 9.68 |
| 961774 | BaNaSb | Sb | Ba | Na | 0.796 | 2.17 |  | 1206717 | LaBiPd | Pd | La | Bi | 0.696 | 4.35 |
| 962063 | LaMgTl | Tl | La | Mg | 0.731 | 6.26 |  | 1206719 | NdNiBi | Ni | Nd | Bi | 0.665 | 5.25 |
| 962068 | CaMgSn | Sn | Ca | Mg | 0.716 | 4.19 |  | 1206720 | PrBiPd | Pd | Pr | Bi | 0.690 | 4.19 |
| 962069 | LiAgTe | Te | Li | Ag | 0.664 | 3.38 |  | 1206744 | TmBiPd | Pd | Tm | Bi | 0.669 | 6.28 |
| 962078 | CaCdSi | Si | Ca | Cd | 0.670 | 3.39 |  | 1206953 | ErBiPd | Pd | Er | Bi | 0.670 | 6.53 |
| 1008624 | YBiPd | Pd | Y | Bi | 0.674 | 4.24 |  | 1206989 | TbBiPt | Pt | Tb | Bi | 0.677 | 5.90 |
| 1008680 | TiGePt | Pt | Ti | Ge | 0.600 | 19.40 |  | 1206992 | LuBiPt | Pt | Lu | Bi | 0.668 | 5.77 |
| 1008858 | NdBiPd | Pd | Nd | Bi | 0.687 | 4.59 |  | 1207057 | TmBiPt | Pt | Tm | Bi | 0.670 | 5.94 |
| 1009006 | LiCdAs | As | Li | Cd | 0.634 | 16.44 |  | 1207082 | LiMgSb | Sb | Li | Mg | 0.667 | 14.63 |
| 1009132 | HoBiPd | Pd | Ho | Bi | 0.672 | 6.78 |  | 1207177 | TbBiPd | Pd | Tb | Bi | 0.675 | 6.93 |
| 1009543 | DyBiPd | Pd | Dy | Bi | 0.673 | 6.93 |  | 1207185 | LuBiPd | Pd | Lu | Bi | 0.666 | 5.75 |
| 1018118 | TmSbPd | Pd | Tm | Sb | 0.656 | 7.66 |  | 1216635 | TiSnIr | Ir | Ti | Sn | 0.622 | 3.90 |
| 1018135 | LiCdAs | As | Li | Cd | 0.634 | 16.49 |  | 1225491 | DySbPd | Pd | Dy | Sb | 0.661 | 7.67 |
| 1018139 | HoNiBi | Ni | Ho | Bi | 0.647 | 9.44 |  |  |  |  |  |  |  |  |

[1] Togo, A., Chaput, L., & Tanaka, I., Distributions of phonon lifetimes in Brillouin zones, Phys. Rev. B, 91, 094306 (2015).

Table S2

List of parameters for machine learning used for thermal conductivity prediction. It is listed by various combinations of atomic mass (*m*_1_, *m*_2_, *m*_3_) and atomic radius (*r*_1_, *r*_2_, *r*_3_) of elementals at 4*c*, 4*a*, 4*b* site. The lattice parameter, *x*_55_, uses the predicted values of the lattice parameters obtained by the multiple linear regression in Fig. 2(b2).

|  | Parameters |  |  | Parameters |  |  | Parameters |
| --- | --- | --- | --- | --- | --- | --- | --- |
| *x*_1_ | $m_{1}$ |  | *x*_20_ | $\sqrt{m_{2}}$ |  | *x*_39_ | ${x_{33}}^{2}$ |
| *x*_2_ | $m_{2}$ |  | *x*_21_ | $\sqrt{m_{3}}$ |  | *x*_40_ | ${x_{34}}^{2}$ |
| *x*_3_ | $m_{3}$ |  | *x*_22_ | $\sqrt{r_{1}}$ |  | *x*_41_ | ${x_{35}}^{2}$ |
| *x*_4_ | $r_{1}$ |  | *x*_23_ | $\sqrt{r_{2}}$ |  | *x*_42_ | ${x_{30}}/3-r_{1}$ |
| *x*_5_ | $r_{2}$ |  | *x*_24_ | $\sqrt{r_{3}}$ |  | *x*_43_ | ${x_{30}}/3-r_{2}$ |
| *x*_6_ | $r_{3}$ |  | *x*_25_ | ${m_{2}}/{m_{1}}$ |  | *x*_44_ | ${x_{30}}/3-r_{3}$ |
| *x*_7_ | ${m_{1}}^{2}$ |  | *x*_26_ | ${r_{3}}/{m_{1}}$ |  | *x*_45_ | $\left\vert x_{42} \right\vert$ |
| *x*_8_ | ${m_{2}}^{2}$ |  | *x*_27_ | ${r_{2}}/{r_{1}}$ |  | *x*_46_ | $\left\vert x_{43} \right\vert$ |
| *x*_9_ | ${m_{3}}^{2}$ |  | *x*_28_ | ${r_{3}}/{r_{1}}$ |  | *x*_47_ | $\left\vert x_{44} \right\vert$ |
| *x*_10_ | ${r_{1}}^{2}$ |  | *x*_29_ | $m_{1}+m_{2}+m_{3}$ |  | *x*_48_ | ${x_{42}}^{2}$ |
| *x*_11_ | ${r_{2}}^{2}$ |  | *x*_30_ | $r_{1}+r_{2}+r_{3}$ |  | *x*_49_ | ${x_{43}}^{2}$ |
| *x*_12_ | ${r_{3}}^{2}$ |  | *x*_31_ | $\left( \left( {m_{1}}^{2}+{m_{2}}^{2}+{m_{3}}^{2} \right)/3 \right)^{2}$ |  | *x*_50_ | ${x_{44}}^{2}$ |
| *x*_13_ | ${m_{1}}^{3}$ |  | *x*_32_ | $\left( \left( {r_{1}}^{2}+{r_{2}}^{2}+{r_{3}}^{2} \right)/3 \right)^{2}$ |  | *x*_51_ | ${r_{1}}^{2}+{r_{2}}^{2}$ |
| *x*_14_ | ${m_{2}}^{3}$ |  | *x*_33_ | ${x_{29}}/3-m_{1}$ |  | *x*_52_ | ${r_{1}}^{2}+{r_{3}}^{2}$ |
| *x*_15_ | ${m_{3}}^{3}$ |  | *x*_34_ | ${x_{29}}/3-m_{2}$ |  | *x*_53_ | $\sqrt{x_{51}}$ |
| *x*_16_ | ${r_{1}}^{3}$ |  | *x*_35_ | ${x_{29}}/3-m_{3}$ |  | *x*_54_ | $\sqrt{x_{52}}$ |
| *x*_17_ | ${r_{2}}^{3}$ |  | *x*_36_ | $\left\vert x_{33} \right\vert$ |  | *x*_55_ | Predicted lattice parameter |
| *x*_18_ | ${r_{3}}^{3}$ |  | *x*_37_ | $\left\vert x_{34} \right\vert$ |  |  |  |
| *x*_19_ | $\sqrt{m_{1}}$ |  | *x*_38_ | $\left\vert x_{35} \right\vert$ |  |  |  |
